# *Mycobacterium leprae* in Nine-Banded Armadillos (*Dasypus novemcinctus*), Ecuador

**DOI:** 10.3201/eid3012.231143

**Published:** 2024-12

**Authors:** Daniel Romero-Alvarez, Manuel Calvopiña, Emily Cisneros-Vásquez, Daniel Garzon-Chavez, Alaine K. Warren, Lauren S. Bennett, Ritika R. Janapati, Carlos Bastidas-Caldes, Melanie Cabezas-Moreno, Jacobus H. de Waard, Daniela Silva-Martinod, Roxane Schaub, Mary Jackson, A. Townsend Peterson, Charlotte Avanzi

**Affiliations:** Universidad Internacional SEK, Quito, Ecuador (D. Romero-Alvarez); University of Kansas, Lawrence, Kansas, USA (D. Romero-Alvarez, A.T. Peterson); Universidad de las Américas, Quito (M. Calvopiña, E. Cisneros-Vásquez, C. Bastidas-Caldes, J.H. de Waard, D. Silva-Martinod); Universidad San Francisco de Quito, Quito (D. Garzon-Chavez); Colorado State University, Fort Collins, Colorado, USA (A.K. Warren, L.S. Bennett, R.R. Janapati, M. Jackson, C. Avanzi); Agrocalidad, Quito (M. Cabezas-Moreno); Centre Hospitalier de Cayenne, Cayenne, French Guiana (R. Schaub)

**Keywords:** Mycobacterium leprae, bacteria, zoonoses, nine-banded armadillos, Dasypus novemcinctus, leprosy, Hansen disease, environmental clusters, Ecuador, tuberculosis and other mycobacteria

## Abstract

We found *Mycobacterium leprae*, the most common etiologic agent of Hansen disease or leprosy, in tissues from 9 (18.75%) of 48 nine-banded armadillos (*Dasypus novemcinctus*) collected across continental Ecuador. Finding evidence of a wildlife reservoir is the first step to recognizing leprosy zoonotic transmission pathway in Ecuador or elsewhere.

The World Health Organization Global Leprosy Strategy targets the long-term goal of leprosy elimination through interruption of disease transmission (*1*). One factor that can impair that goal is environmental or animal reservoirs that contribute to persistence of the *Mycobacterium leprae* bacteria and potential spillover into the human population. The Strategy acknowledges that *M. leprae* zoonotic transmission exists but with a lower risk and highly localized in North America (*1*), possibly because of a lack of research on new and existing animal reservoirs in other locations.

*M. leprae*, the main causative agent of leprosy, has a broad range of animal hosts, including wild armadillos (*Dasypus* spp.) in the Americas, red squirrels (*Sciurus vulgaris*) in the British Isles. and nonhuman primates in the Philippines and Africa (*2*). Armadillos are a family of medium-sized mammals, belonging to the Xenarthrans, which also includes sloths and anteaters (*3*). At least 20 armadillo species have been recognized (*3*). The Cingulata order encompasses >9 *Dasypus* species, including the nine-banded armadillo (*D. novemcinctus*), considered the main *M. leprae* reservoir in the Americas (*2*).

Ecuador, located in northwestern South America, is a medium-income economy nation with ≈18 million inhabitants (*4*). Officially, Ecuador eliminated leprosy as a public health threat, which means incidence is <1 new case/10,000 inhabitants; only 41 new leprosy cases were registered in 2022 (*5*). Scientific literature on leprosy in Ecuador is scarce; nonetheless, the Ministry of Public Health suggests a higher disease incidence across the country (*6*). Armadillos are found throughout Ecuador and are valued as a protein source and a cultural item in many rural settings (*7*). In view of the uncertain epidemiologic landscape of leprosy in Ecuador and the occurrence of a possible animal reservoir in the country, we investigated *M. leprae* infection in armadillos in Ecuador.

## The Study

We gathered tissue samples from 45 armadillos via local hunters who use the mammal as a protein source for their families and communities. The Instituto Nacional de Biodiversidad (Quito, Ecuador) also donated 3 additional samples stored in 70% ethanol, for a total of 48 armadillos. We performed tissue collection according to a protocol approved by the Ministerio del Ambiente, Agua y Transición Ecológica, as part of the Genetic Resources Access Framework contract (contract no. MAATE-DBI-CM-2021-0172). We established definitive armadillo species identification by morphological features, known geographic distributions, and molecular diagnosis (Appendix 1). We processed >2 tissues from 36 armadillos and only 1 tissue for the other 12; we examined each tissue >2 times (Appendix 1). We performed DNA extraction and pathogen identification via real-time quantitative PCR (qPCR) using previously well-established primers and protocols (*8*,*9*) (Appendix 1). We considered a sample positive for *M. leprae* or *M. lepromatosis* only if 2 independent qPCR runs yielded a cycle threshold (Ct) <35 (*9*,*10*).

We processed a total of 84 armadillo tissue samples (Appendix 2), including 38 (45.24%) liver, 26 (30.95%) spleen, and 10 (11.9%) muscle samples (Table). We identified *M. leprae* DNA in 13 (15.48%) samples, mostly from liver (n = 8/38 [21.05%]) and spleen (n = 4/26 [15.38%]) (Table). For 3 armadillos with varying results between tissues, the liver was the source of positivity (Appendix 2). All 84 tissue samples were negative for *M. lepromatosis* according to our protocols.

**Table:** Characteristics of animals and samples tested in a study of *Mycobacterium leprae* in nine-banded armadillos (*Dasypus novemcinctus*), Ecuador*

Categories	No. (%)	*M. leprae*–positive, no. (%)
Tissue samples		
Liver	38 (45.24)	8 (21.05)
Spleen	26 (30.95)	4 (15.38)
Muscle	10 (11.90)	1 (10)
Heart	3 (3.57)	0 (0)
Kidney	3 (3.57)	0 (0)
Lung	3 (3.57)	0 (0)
Ear	1 (1.19)	0 (0)
Total	84 (100)	13 (15.48)
Armadillo species		
* Dasypus novemcinctus*	40 (83.33)	9 (22.50)
*Dasypus* spp.	6 (12.5)	0 (0)
* D. pastasae*	1 (2.08)	0 (0)
* Cabassous centralis*	1 (2.08)	0 (0)
Total	48 (100)	9 (18.75)
Provinces		
Esmeraldas	10 (20.83)	1 (10)
Sucumbíos	9 (18.75)	1 (11.11)
Guayas	7 (14.58)	0 (0)
Santo Domingo de los Tsáchilas	7 (14.58)	3 (42.86)
Morona Santiago	4 (8.3)	0 (0)
Pastaza	3 (6.25)	1 (33.33)
Imbabura	2 (4.17)	1 (50)
Manabí	2 (4.17)	1 (50)
Cañar	1 (2.08)	1 (100)
Cotopaxi	1 (2.08)	0 (0)
Los Ríos	1 (2.08)	0 (0)
No information	1 (2.08)	0 (0)
Total	48 (100)	9 (18.75)

*A total of 84 tissue samples corresponding to 48 individual armadillos from 4 species were tested for *Mycobacterium leprae*. Percentage of *M. leprae* positives is calculated with the total sampling of each row. No samples were positive for *M. lepromatosis*.

The 48 individual armadillos belonged to 4 different species: 40 (83.33%) were *D. novemcinctus*, 6 (12.5%) *Dasypus* spp. (not identified to species), 1 (2.08%) *D. pastasae*, and 1 (2.08%) *Cabassous centralis* (Table; Figure). We detected *M. leprae* in 9 *D. novemcinctus* armadillos, for an overall prevalence of 18.75%. Ct values were 26.01–33.66 (Table, Figure; Appendix 2). Most (20.83%, 10/48) armadillos were collected in the Esmeraldas province along the coast, among which only 1 (10%) *D. novemcinctus* armadillo was *M. leprae–*positive (Figure, Table). We observed the highest prevalence (42.86%) of infected armadillos in Santo Domingo de los Tsáchilas, in the northwest, where 3 of 7 animals were *M. leprae*–positive (Figure).

**Figure:**
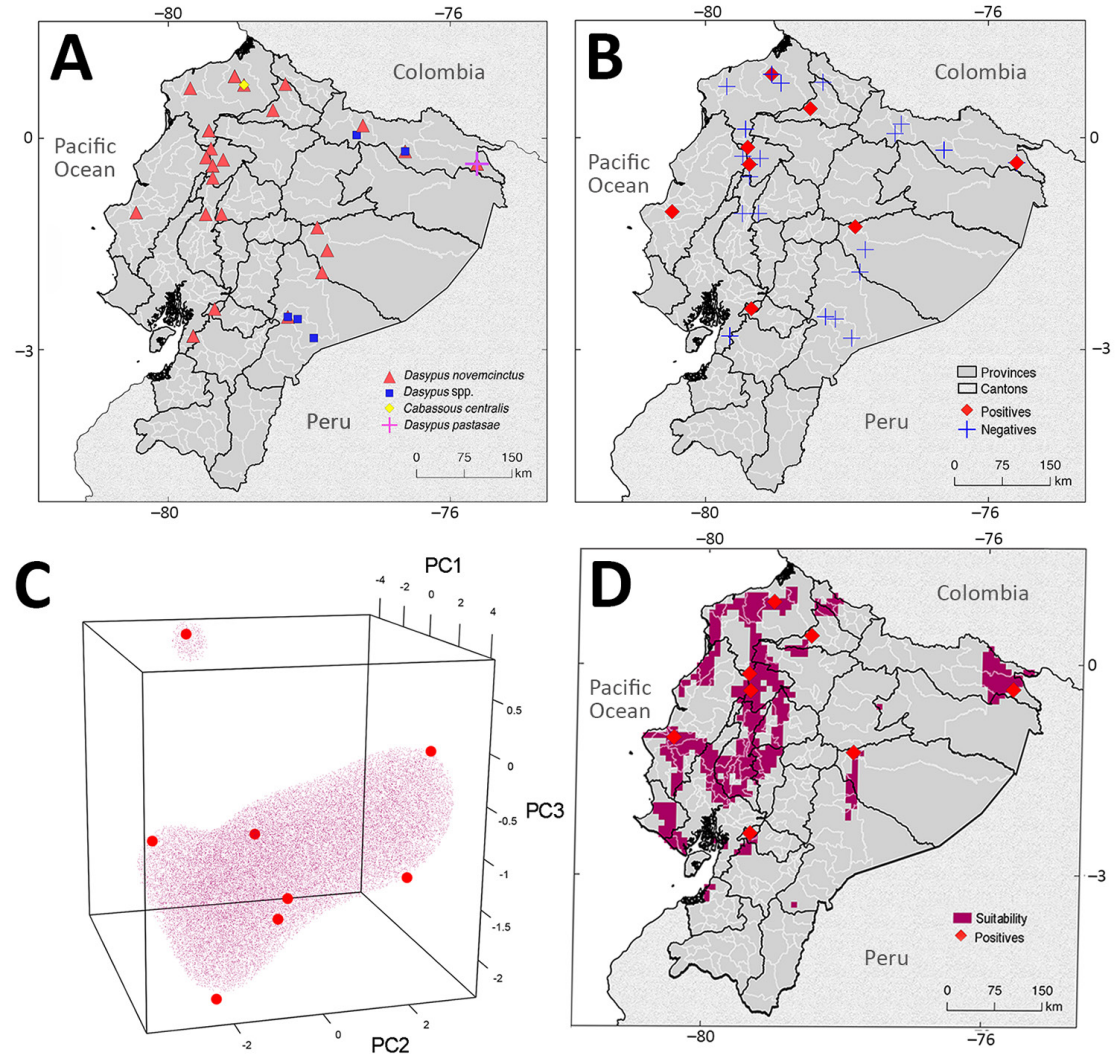
Locations of and geographic range *Mycobacterium leprae* detections in a study of *M. leprae* in nine-banded armadillos (*Dasypus novemcinctus*), Ecuador. A) Locations of armadillo collections and species identified. B) Locations from which *M. leprae­*–positive armadillos samples collected. In southern Santo Domingo de los Tsáchilas, >1 armadillo was collected (Appendix 2). No samples were positive for *M. lepromatosis*. C, D) Vector machine hypervolume and its projected geography. C) One-class support vector machine hypervolume with enclosed regions of environmental similarity to areas with *M. leprae* detections (red points); D) map with projected geography for *M. leprae* detections. Latitude and longitude are shown at edges. Mapping developed with the information available in Appendix 2. PC, principal component (see Appendix 1).

To characterize potential clusters of infected *D. novemcinctus* armadillos in Ecuador, we used the localities of the 9 *M. leprae–*positive armadillos to develop a species distribution model based on 1-class support vector machine hypervolumes (*11*) and 20 environmental predictors (Figure, panel C; Appendix 1 Figure 1). The subtropical region of Ecuador, west of the Andes mountains, had the highest concentration of environments like those with *M. leprae*–positive detections (Figure, panel D). Specifically, Esmeraldas, Los Ríos, Santo Domingo de los Tsáchilas, Santa Elena, northern Bolívar and Guayas, and southern Manabí are regions with environmental similarities to locales where infected *D. novemcinctus* armadillo were found (Figure).

## Conclusions

The canon of leprosy transmission has been actively rewritten in the past 2 decades (*2*). Confirmation of zoonotic *M. leprae* transmission in the United States (*7*) prompted a series of studies to evaluate the spread of leprosy bacilli in the *D. novemcinctus* armadillo across its range in the Americas (Appendix 1 Figure 2). Our research demonstrated that nine-banded armadillos from the 3 continental regions of Ecuador host *M. leprae* with an 18.75% prevalence (Table; Figure). Detection of bacilli in wild armadillos is the first step in evaluating leprosy as a zoonotic pathogen in Ecuador. All 84 tissues examined were negative for *M. lepromatosis*, in agreement with previous results for other mammals in Europe and Mexico (*12*,*13*) and for armadillo specimens from across the Americas (*8*).

One limitation of our study was that our sampling scheme depended on local hunters who collect armadillos; thus, systematic sampling representing specific ecologic regions was unfeasible. Moreover, sampling scope was initially limited to tissues in ethanol, preventing serology and histopathology investigations. Nevertheless, we were able to collect tissues from across the country and observed consistency in the molecular detection of *M. leprae* with multiple rounds of qPCR in DNA extracted from various tissues from the same armadillo (Table; Appendix 2). Of note, a Ct value <35 in 2 independent qPCR rounds per tissue as criteria for *M. leprae* positivity is conservative, yet informative of the pathogen in nine-banded armadillos in the country.

Ecuador hosts at least 5 armadillo species: *Cabassous centralis*, *C. unicinctus*, *Priodontes maximus*, *D. pastasae*, and *D. novemcintus* (*14*). *D. novemcintus* was the most common (83.33%) armadillo species in our sampling and the only *M. leprae*–positive species (Table). We identified 3 other armadillo species, but all were *M. leprae*–negative (Figure, Table). Apart from *D. novemcinctus*, armadillo species in which *M. leprae* has been identified beyond Ecuador include *Euphractus sexcinctus, Dasypus* spp. nov., and *D. sabanicola*. Moreover, *D. septemcinctus* armadillos have been shown to be susceptible to *M. leprae* laboratory infections (*15*).

Species distribution models have seldom been used to characterize leprosy geographic range. Given the epidemiologic tenets of *M. leprae*, including long incubation period and human-to-human transmission, data for those models is difficult to obtain. Moreover, information on *M. leprae* prevalence in armadillos is either overrepresented as in the southern United States, or scant and dispersed as in the rest of the Americas (*2*) (Appendix 1 Figure 4). Thus, by leveraging our *M. leprae*–positive armadillo detections across the landscape of Ecuador, our model depicted clusters of environmental similarity. Considering the inherent challenges to collecting and studying armadillos (*3*), our model could be used to optimize future expedition sampling. 

In conclusion, presence of a nonhuman *M. leprae* host carrier, the nine-banded armadillo, is likely to contribute directly or indirectly to the human leprosy incidence in Ecuador and other countries and will likely impair long-term goals of disease elimination. However, the detection of *M. leprae* in armadillos from Ecuador should exemplify how continued sampling and surveillance in wildlife can avert future zoonotic infections.

Appendix 1 Additional information on methods for the experiment of *Mycobacterium leprae* in nine-banded armadillos (*Dasypus novemcinctus*), Ecuador.

Appendix 2 Raw data used for modeling of *Mycobacterium leprae* in nine-banded armadillos (*Dasypus novemcinctus*), Ecuador.
