# Supplementary material for: Mycobacterium leprae in Nine-Banded Armadillos (Dasypus novemcinctus), Ecuador
Source: Emerg Infect Dis. 2024 Dec;30(12):2629–32. doi: 10.3201/eid3012.231143 (PMC11616649; doi:10.3201/eid3012.231143)
Supplement: Appendix 1 — Additional information on methods for the experiment of Mycobacterium leprae in nine-banded armadillos (Dasypus novemcinctus), Ecuador. [file 23-1143-Techapp-s1.pdf]

Article DOI: https://doi.org/10.3201/eid3012.231143
EID cannot ensure accessibility for supplementary materials supplied by authors. Readers who have difficulty accessing supplementary content should contact the authors for assistance.

Mycobacterium leprae in Nine-Banded Armadillos (*Dasypus novemcinctus*), Ecuador

Appendix 1

Supplementary Methods

Detailed Methodology for DNA Extraction and Real-Time PCR

Detection of *Mycobacterium leprae* and *M. lepromatosis*

Two armadillo tissues were processed for 36 individuals and only one for 12 armadillos. For each tissue, at least two rounds of molecular diagnosis were applied. DNA extraction was performed using the QIAMP UCP Pathogen Minikit (Qiagen, Germany) following manufacturer instructions with minimal modifications. Briefly, ≈50–200mg of tissue were collected and rehydrated in phosphate buffer saline. After overnight incubation, the tissue was transferred to bead-beating tubes CKMix (Bertin Corp., Rockville, U.S.) and submitted to mechanical lysis for 30sec at 7200rpm in a Precellys® 24 instrument. Chemical lysis using 40µL of proteinase K (Qiagen, Germany) in ATL buffer (volume of 500µL final) was followed by incubation at 56°C until complete digestion of the tissue or overnight at 37°C. After a brief spin-down, the content of the tube was transferred to a new bead-beating tube containing 0.1mm zirconia bead. Mechanical lysis was then performed with three rounds of bead beating at 6800rpm for 30sec with five minutes on ice between rounds. The tube was then centrifuged at 10,000 g for one minute, and the supernatant was transferred to a new 2mL Eppendorf tube containing 200µL of APL2 buffer (Qiagen, Germany). After an incubation of 10min at 70°C the DNA was precipitated using 200 µL of absolute ethanol and purified on QIAmp UCP Pathogen Mini silica column as per manufacturer instructions.

All extracted samples were processed via quantitative real-time PCR (qPCR) targeting the repetitive element RLEP for *M. leprae* and the repetitive element RLPM for *M. lepromatosis* as previously described (*1*,*2*). We used 2uL of DNA to a PCR volume of 20uL containing 10uL SsoAdvanced Universal Probes Supermix (Biorad, CA, U.S.), 900 nM of each forward and reverse primers, and 250 nM of the corresponding hydrolysis probe. Initial denaturation for amplification state was done with 95°C for 10 min and 60°C for 1 minute for 40 cycles. We analyzed data using the CFX Maestro Software (BioRad, CA, U.S.). A sample was considered positive for *M. leprae* or *M. lepromatosis* only if two independent qPCR runs per tissue yielded readable cycle thresholds (Cts) below a value of 35 (i.e., Ct<35).

Positive controls included 2µL of a plasmid containing the RLEP region of *M. leprae* (*2*) and 2µL of *M. lepromatosis* (DNA diluted 1:100 from the stock received) donated by Dr. Ramanuj Lahiri from the National Hansen's Disease Program. Molecular grade water served as a negative control in each PCR run.

Molecular Identification of Armadillo Species

Extractions of DNA armadillo samples were used for standard PCR amplification of the cytochrome oxidase subunit I (COI) (*3*), amplifying a universal mitochondrial marker obtaining a 710bp fragment. Amplification was performed using 100ng of DNA and 25µL of DreamTaq mastermix (Thermo Fisher, MA, U.S.), 2.5 µL of forward primer at 5 µM (LCO1490–3′-GGTCAACAAATCATAAAGATATTGG-5′) and 2.5µL of reverse primer at 5 µM (HCO2198–3′-TAAACTTCAGGGTGACCAAAAAATCA-5′).

PCR products were subjected to 1 min at 95°C followed by 40 cycles of 30 sec at 95°C, 30 sec at 52°C, and 1 min at 68°C, coupled with a final extension of 10 min at 68°C. Positive control for this step included 2µL of DNA from an armadillo specimen collected in a previous study (U.S.-am-109) (*4*); molecular-grade water served as a negative control in each PCR run. Amplified DNA was visualized using the Bio-Rad Gel Doc EZ Electrophoresis Unit in a 1% agarose gel at 120V and SybrSafe (Thermo Fisher, MA, U.S.). Amplicons were purified using ExoSap IT PCR product cleanup reagent and sent for Sanger sequencing via Genewiz. Sequences were compared with published armadillo sequences available in NCBI using Geneious Prime® 2023.2.1. Sequences have been uploaded to GenBank with accession number 13769795.

Detailed Methodology for Development Species Distribution Models

Species distribution models are relevant tools for infectious disease mapping in ecologic and biogeographic contexts with applications across different pathogen systems (*5*). These models relate geographic localities at which a particular pathogen has been detected with environmental information to infer geographies with similar conditions (*6*). Pathogens with the capacity to endure in the environment depend on abiotic conditions for survival, these conditions can be mapped as demonstrated for *Bacillus cereus* biovar *anthracis*, causing anthrax in wildlife in Africa (*7*), or *Burkholderia pseudomallei*, causing melioidosis (*8*). Classical experiments on *M. leprae* environmental tolerance have suggested that the pathogen is able to survive in wet soil for up to 46 days, which may be related to its capacity to persist inside amoeba cysts (*9*,*10*). Moreover, studies demonstrating *M. leprae* viability in soil and water samples (*11*) suggest that armadillos might be acquiring the infection directly from environmental sources

Modeling Scheme

Species distribution models were developed using one-class support vector machine hypervolumes (OC-SVM (*12*);). OC-SVM builds a hyperellipse in the environmental space around occurrences, and trims it based on established parameters to suggest environmental similarities across the study area (*12*). Outputs from OC-SVM were binarized automatically by the algorithm and interpreted as clusters of environmental similarity across Ecuadorian landscape to depict areas of suitability for *Dasypus novemcinctus* infected with *M. leprae* (Figure main text).

Environmental Predictors

Considering known details of *M. leprae* requirements for survival (*9*,*13*), we constrained our environmental predictors to those representing temperature, humidity, soil chemical properties, and vegetation. For temperature and humidity, we used the open-access repository of bioclimatic variables MERRAclim, which uses satellite imagery to derive summaries of worldwide climatic conditions (*14*). The four bioclimatic variables combining temperature and humidity (e.g., BIO8 = mean temperature of most humid quarter) are known for creating spatial artifacts and were excluded from model development (*15*). MERRAclim variables were downloaded at 5′, ≈8 km at the Equator; we used the database from 2000–2010 for temporal resolution. To represent soil chemistry, we used variables from the SoilGrids repository, at a

resolution of 250m using a combination of field and satellite data (*16*). Specifically, we included summaries of cation exchange capacity (CECSOL), organic soil carbon (ORCDRC), water pH (PHIHOX), and nitrogen at one depth (0–5 cm). Finally, we used the Enhanced Vegetation Index (EVI) as a proxy of vegetation availability in the study area (*17*). We collected satellite images from 2010–2021 (version 6) from the Moderate Resolution Imaging Spectroradiometer (MODIS) instrument from the TERRA satellite (i.e., MOD13Q1, 16 day composites, 250m resolution (*18*);), and averaged them to obtain a summary of vegetation characteristics via Google Earth Engine (*19*). All variables were resampled to match the resolution of MERRAclim (i.e., 5′). To avoid overfitted model outputs owing to multicollinearity (*20*), we applied a singular value decomposition principal component analysis (PCA) and used the PCs summarizing >85% of the variance as final predictors for model building (i.e., PCs 1–3). Raster data collection and manipulation were performed using the 'raster' and 'kuenm' packages in R (*21*,*22*).

References

1. Sharma R, Singh P, Pena M, Subramanian R, Chouljenko V, Kim J, et al. Differential growth of *Mycobacterium leprae* strains (SNP genotypes) in armadillos. Infect Genet Evol. 2018;62:20–6. PubMed https://doi.org/10.1016/j.meegid.2018.04.017

2. Avanzi C, Lécorché E, Rakotomalala FA, Benjak A, Rapelanoro Rabenja F, Ramarozatovo LS, et al. Population genomics of *Mycobacterium leprae* reveals a new genotype in Madagascar and the Comoros. Front Microbiol. 2020;11:711. PubMed https://doi.org/10.3389/fmicb.2020.00711

3. Folmer O, Black M, Hoeh W, Lutz R, Vrijenhoek R. DNA primers for amplification of mitochondrial cytochrome c oxidase subunit I from diverse metazoan invertebrates. Mol Mar Biol Biotechnol. 1994;3:294–9. PubMed

4. Romero-Alvarez D, Garzon-Chavez D, Jackson M, Avanzi C, Peterson AT. *Mycobacterium leprae* in armadillo tissues from museum collections, United States. Emerg Infect Dis. 2023;29:622–6. PubMed https://doi.org/10.3201/eid2903.221636

5. Escobar LE, Craft ME. Advances and limitations of disease biogeography using ecological niche modeling. Front Microbiol. 2016;7:1174. PubMed https://doi.org/10.3389/fmicb.2016.01174

6. Peterson AT, Soberón J, Pearson RG, Anderson RP, Martínez-Meyer E, Nakamura M, et al. Ecological Niches and Geographic Distributions. New Jersey: Princeton University Press; 2011.

7. Romero-Alvarez D, Peterson AT, Salzer JS, Pittiglio C, Shadomy S, Traxler R, et al. Potential distributions of *Bacillus anthracis* and *Bacillus cereus* biovar *anthracis* causing anthrax in Africa. PLoS Negl Trop Dis. 2020;14:e0008131. PubMed https://doi.org/10.1371/journal.pntd.0008131

8. Limmathurotsakul D, Golding N, Dance DAB, Messina JP, Pigott DM, Moyes CL, et al. Predicted global distribution of *Burkholderia pseudomallei* and burden of melioidosis. Nat Microbiol. 2016;1:15008. PubMed https://doi.org/10.1038/nmicrobiol.2015.8

9. Desikan KV, Sreevatsa. Effect of adverse environmental conditions on *Mycobacterium leprae.* Indian J Clin Biochem. 1997;12(Suppl 1):89–92. PubMed https://doi.org/10.1007/BF02873071

10. Wheat WH, Casali AL, Thomas V, Spencer JS, Lahiri R, Williams DL, et al. Long-term survival and virulence of *Mycobacterium leprae* in amoebal cysts. PLoS Negl Trop Dis. 2014;8:e3405. PubMed https://doi.org/10.1371/journal.pntd.0003405

11. Turankar RP, Lavania M, Darlong J, Siva Sai KSR, Sengupta U, Jadhav RS. Survival of *Mycobacterium leprae* and association with *Acanthamoeba* from environmental samples in the inhabitant areas of active leprosy cases: A cross sectional study from endemic pockets of Purulia, West Bengal. Infect Genet Evol. 2019;72:199–204. PubMed https://doi.org/10.1016/j.meegid.2019.01.014

12. Blonder B, Morrow CB, Maitner B, Harris DJ, Lamanna C, Violle C, et al. New approaches for delineating n-dimensional hypervolumes. Methods Ecol Evol. 2018;9:305–19. https://doi.org/10.1111/2041-210X.12865

13. Turankar RP, Lavania M, Singh M, Siva Sai KSR, Jadhav RS. Dynamics of *Mycobacterium leprae* transmission in environmental context: deciphering the role of environment as a potential reservoir. Infect Genet Evol. 2012;12:121–6. PubMed https://doi.org/10.1016/j.meegid.2011.10.023

14. Vega GC, Pertierra LR, Olalla-Tárraga MÁ. MERRAclim, a high-resolution global dataset of remotely sensed bioclimatic variables for ecological modelling. Sci Data. 2017;4:170078. PubMed https://doi.org/10.1038/sdata.2017.78

15. Booth TH. Checking bioclimatic variables that combine temperature and precipitation data before their use in species distribution models. Austral Ecol. 2022;47:1506–14. https://doi.org/10.1111/aec.13234

16. Hengl T, Mendes de Jesus J, Heuvelink GBM, Ruiperez Gonzalez M, Kilibarda M, Blagotić A, et al. SoilGrids250m: global gridded soil information based on machine learning. PLoS One. 2017;12:e0169748. PubMed https://doi.org/10.1371/journal.pone.0169748

17. Horning N, Robinson JA, Sterling EJ, Turner W, Spector S. Remote sensing for ecology and conservation: a handbook of techniques. New York: Oxford University Press; 2010.

18. Didan K. MOD13A2 MODros Inf Serv./Terra Vegetation Indices 16-Day L3 Global 1km SIN Grid V006 [Data set]. NASA EOSDros Inf Serv. Land Processes Distributed Active Archive Center. 2015. [cited 2023 Aug 21]. https://doi.org/10.5067/MODIS/MOD13A2.006

19. Gorelick N, Hancher M, Dixon M, Ilyushchenko S, Thau D, Moore R. Google Earth Engine: planetary-scale geospatial analysis for everyone. Remote Sens Environ. 2017;202:18–27. https://doi.org/10.1016/j.rse.2017.06.031

20. Cobos ME, Peterson AT, Osorio-Olvera L, Jiménez-García D. An exhaustive analysis of heuristic methods for variable selection in ecological niche modeling and species distribution modeling. Ecol Inform. 2019;53:100983. https://doi.org/10.1016/j.ecoinf.2019.100983

21. Cobos ME, Peterson AT, Barve N, Osorio-Olvera L. kuenm: an R package for detailed development of ecological niche models using Maxent. PeerJ. 2019;7:e6281. PubMed https://doi.org/10.7717/peerj.6281

22. Hijmans RJ, Van Etten J, Sumner M, Cheng J, Baston D, Bevan A, et al. Package ‘raster’: geographic data analysis and modeling. R package. 2015. [cited 2023 Aug 21]. https://cran.r-project.org/web/packages/raster/raster.pdf

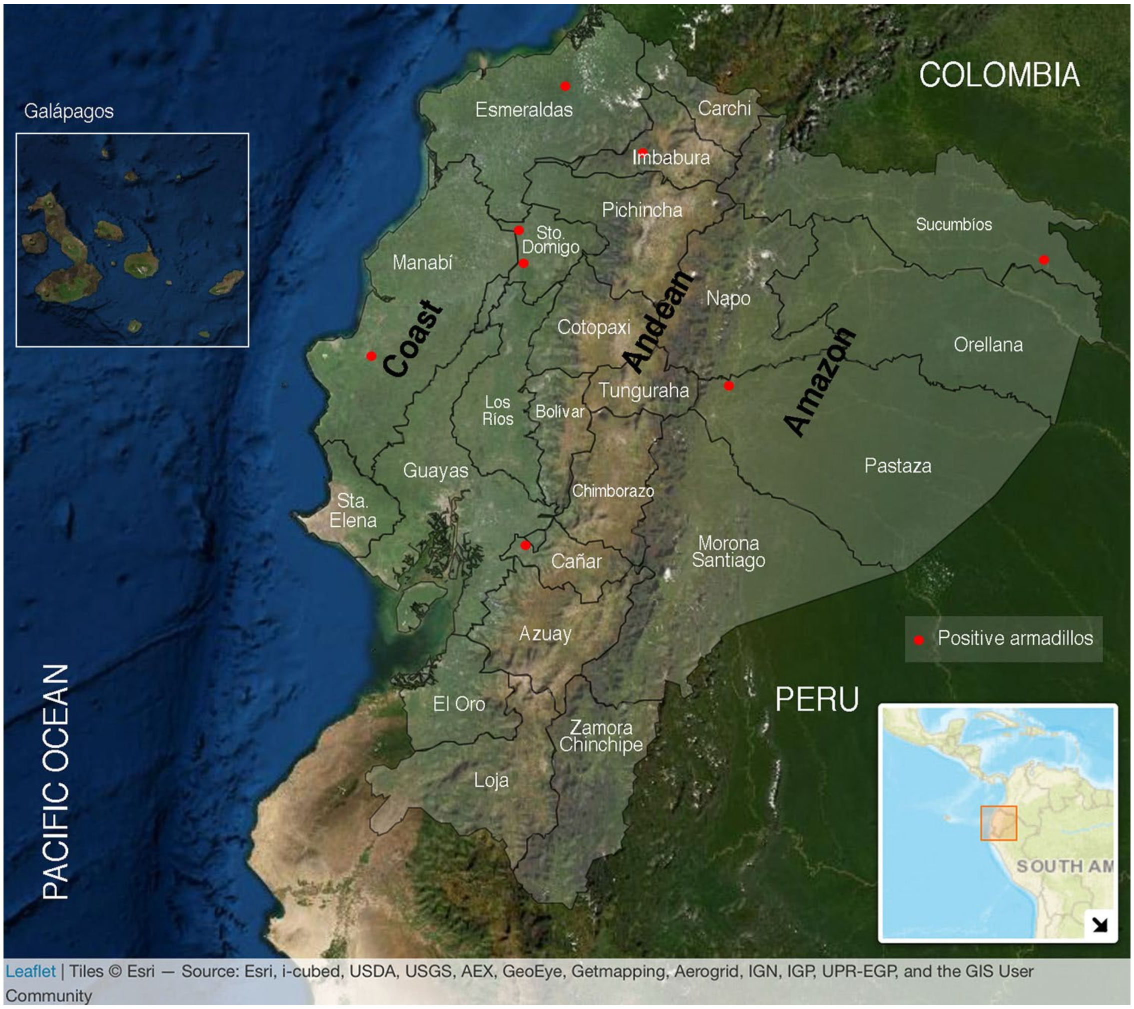

Appendix 1 Figure 1. Physical map of Ecuador depicting provinces as political divisions of the country. Localities with positive *Mycobacterium leprae* detections in nine-banded armadillos are shown as red dots (n = 9); the southern locality at Santo Domingo de los Tsáchilas had 2 armadillos from the same locale). Armadillo species listed in the main text. Map developed with the package 'leaflet' in R (The R Project for Statistical Computing, https://www.r-project.org) and modified with Adobe Photoshop Elements (https://www.adobe.com).

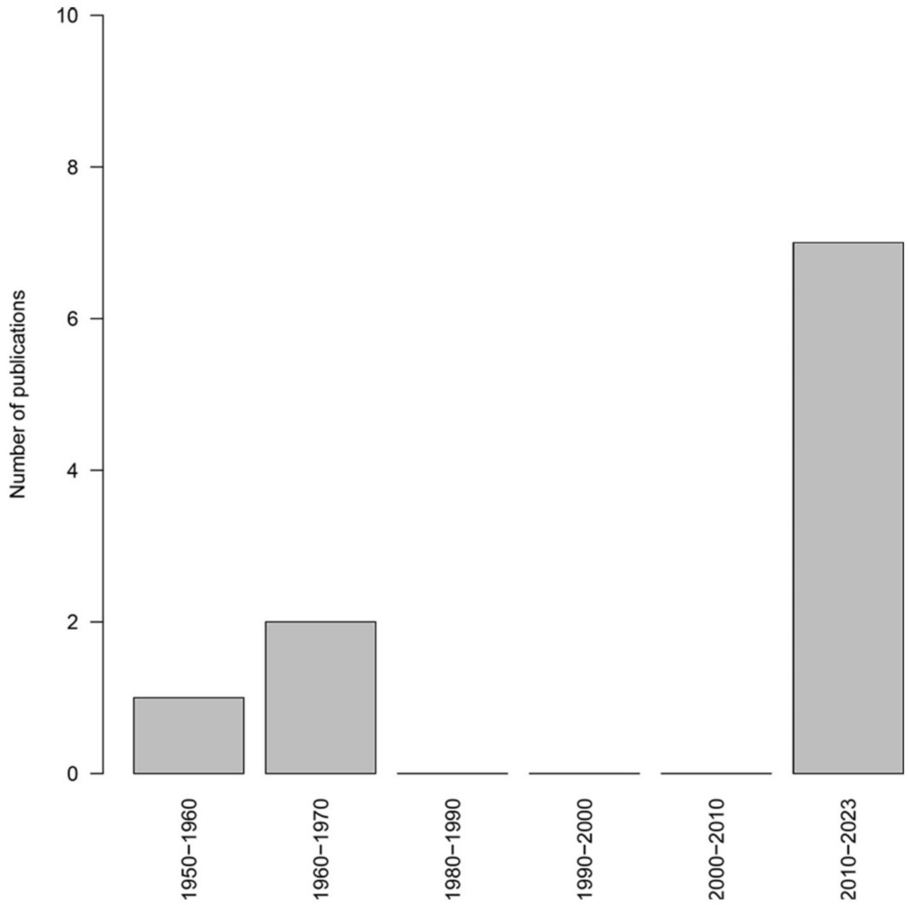

Appendix 1 Figure 2. Frequency of academic publications related to leprosy in Ecuador according to an advanced search in PubMed database performed on June 20, 2023. Search query used: (((((LEPRA[Title/Abstract]) OR (LEPROSY[Title/Abstract])) OR (ENFERMEDAD DE HANSEN[Title/Abstract])) OR (HANSEN'S DISEASE[Title/Abstract])) AND (ECUADOR[Title/Abstract])). We found a total of 10 research papers, the majority published between 2010–2023.

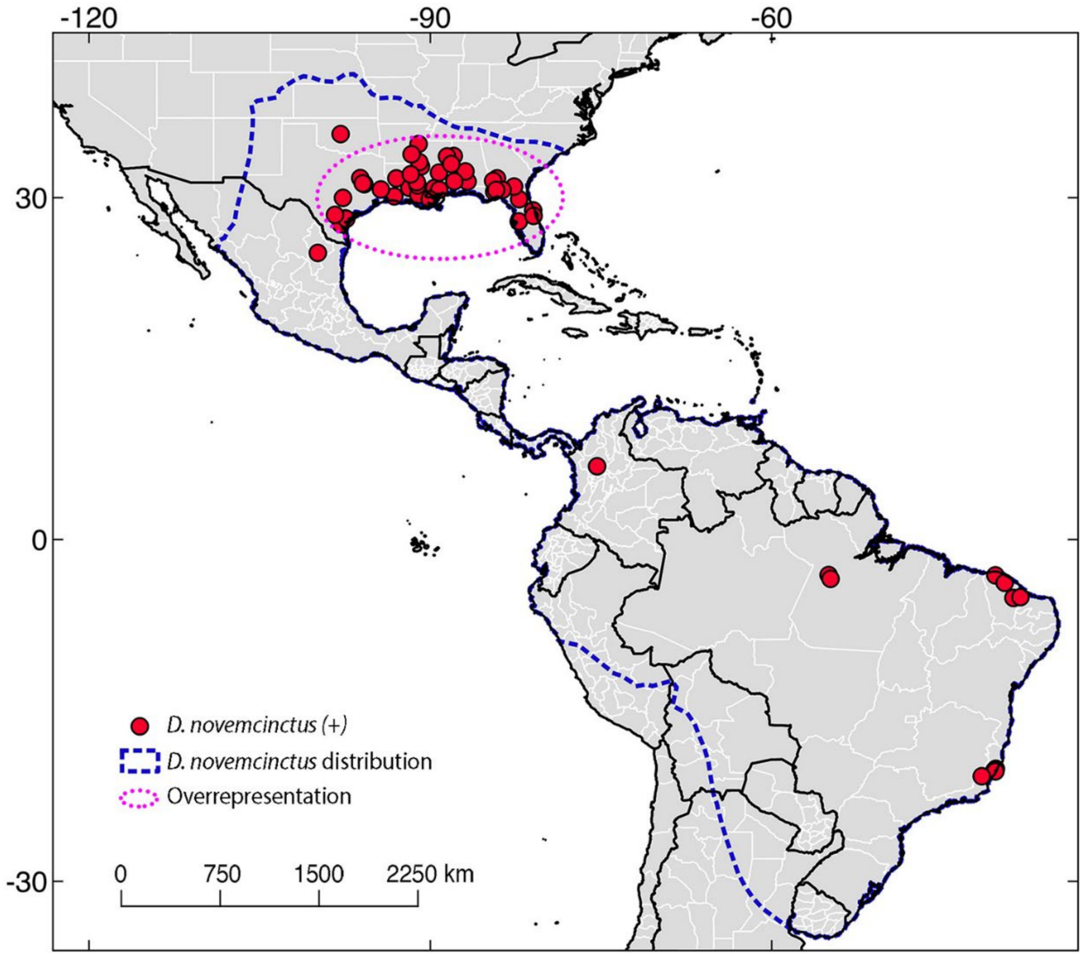

Appendix 1 Figure 3. We collected occurrences of *Dasypus novemcinctus* infected with *Mycobacterium leprae* across the Americas since the 2000s and suggested an updated distribution of *D. novemcinctus* (blue line). Given sampling overrepresentation in the southern United States (pink dotted line) and scant data on *M. leprae* in *D. novemcinctus* elsewhere (red dots), we developed a species distribution model aiming to identify clusters of environmental similarity across the Ecuadorian landscape using the positive *M. leprae* detections of our study (See main text Figure).
